# Inflammatory Biomarkers Differ among Hospitalized Veterans Infected with Alpha, Delta, and Omicron SARS-CoV-2 Variants

**DOI:** 10.3390/ijerph20042987

**Published:** 2023-02-08

**Authors:** Catherine Park, Shahriar Tavakoli-Tabasi, Amir Sharafkhaneh, Benjamin J. Seligman, Bret Hicken, Christopher I. Amos, Andrew Chou, Javad Razjouyan

**Affiliations:** 1 VA’s Health Services Research and Development Service (HSR&D), Center for Innovations in Quality, Effectiveness, and Safety, Michael E. DeBakey VA Medical Center, Houston, TX 77030, USA; 2 Big Data Scientist Training Enhancement Program, VA Office of Research and Development, Washington, DC 20420, USA; 3 VA Quality Scholars Coordinating Center, IQuESt, Michael E. DeBakey VA Medical Center, Houston, TX 77030, USA; 4 Department of Medicine, Baylor College of Medicine, Houston, TX 77030, USA; 5 David Geffen School of Medicine, University of California Los Angeles, Los Angeles, CA 90024, USA; 6 VHA Office of Rural Health, Veterans Rural Health Resource Center, Salt Lake City, UT 84148, USA; 7 George E. Wahlen Department of Veterans Affairs Medical Center, Salt Lake City, UT 84148, USA; 8 Section of Infectious Diseases, Department of Medicine, Baylor College of Medicine, Houston, TX 77030, USA

**Keywords:** inflammatory markers, SARS-CoV-2 variants, alpha, delta, omicron

## Abstract

Mortality due to COVID-19 has been correlated with laboratory markers of inflammation, such as C-reactive protein (CRP). The lower mortality during Omicron variant infections could be explained by variant-specific immune responses or host factors, such as vaccination status. We hypothesized that infections due to Omicron variant cause less inflammation compared to Alpha and Delta, correlating with lower mortality. This was a retrospective cohort study of veterans hospitalized for COVID-19 at the Veterans Health Administration. We compared inflammatory markers among patients hospitalized during Omicron infection with those of Alpha and Delta. We reported the adjusted odds ratio (aOR) of the first laboratory results during hospitalization and in-hospital mortality, stratified by vaccination status. Of 2,075,564 Veterans tested for COVID-19, 29,075 Veterans met the criteria: Alpha (45.1%), Delta (23.9%), Omicron (31.0%). Odds of abnormal CRP in Delta (aOR = 1.85, 95% CI:1.64–2.09) and Alpha (aOR = 1.94, 95% CI:1.75–2.15) were significantly higher compared to Omicron. The same trend was observed for Ferritin, Alanine aminotransferase, Aspartate aminotransferase, Lactate dehydrogenase, and Albumin. The mortality in Delta (aOR = 1.92, 95% CI:1.73–2.12) and Alpha (aOR = 1.68, 95% CI:1.47–1.91) were higher than Omicron. The results remained significant after stratifying the outcomes based on vaccination status. Veterans infected with Omicron showed milder inflammatory responses and lower mortality than other variants.

## 1. Introduction

In late 2021 a new variant of Severe Respiratory Syndrome Coronavirus 2 (SARS-CoV-2) appeared and spread quickly throughout the world [1]. The new variant, named Omicron, was capable of escaping neutralization by antibodies generated by the previous infection and/or vaccination, raising concern for increasing hospitalization and mortality rates [2]. However, hospitalization and mortality rates were lower compared to previous variants [3,4]. Even though the Omicron variant was able to escape neutralizing antibodies to a large extent, pre-existing T and memory B cell immunity are believed to have an important role in limiting the severity of the coronavirus disease of 2019 (COVID-19). However, there is also some evidence to suggest that Omicron is less pathogenic by virtue of decreased fitness to replicate in human lung tissues [5,6]. This decreased pathogenicity may, in turn, result in less immunopathology and less severe COVID-19 disease.

In hospitalized patients with COVID-19 several clinical and laboratory factors have been shown to predict mortality and disease severity [7,8]. Specifically, laboratory markers of inflammation such as C-reactive protein (CRP) and ferritin have shown a strong correlation with disease severity and mortality. Recent data show mortality differences among the SARS-CoV-2 variants dominant in the USA, including Alpha, Delta, and Omicron, with lower mortality in Omicron-related infections. However, it has not been conclusively demonstrated whether the decline in mortality is correlated with the decrease in inflammatory markers or other factors such as vaccination. In this study, we explore if laboratory markers of inflammation differed among the three variants and the differences correlated with the mortality rates. We also were interested to see if such differences are primarily related to vaccination/immunity status or driven by SARS-CoV-2 variants.

## 2. Materials and Methods

We used the veteran health administration (VHA) Corporate Data Warehouse (CDW) [9], and VHA COVID-19 shared data resources [10]. The Research & Development Committee of the Michael E. DeBakey VA Medical Center and Baylor College of Medicine Institutional Review Board (IRB# H-47595) approved our study. 

### 2.1. Cohort

This is a retrospective cohort study that includes all Veterans who tested positive for SARS-CoV-2 between 1 February 2020 and 7 July 2022 and were hospitalized related to COVID-19 infection. The inclusion criteria were the first positive test and hospital admission within 7 days after the SARS-CoV-2 test date or within 15 days prior to the test date [10,11,12]. The exclusion criteria were not being veterans and not being an active VHA healthcare user. We defined the active patients as those veterans that had a primary physician’s visit in the past two years within VHA health care systems. 

### 2.2. Variables

#### 2.2.1. Exposures

The three SARS-CoV-2 variants were determined based on the date of infection after consulting with the infectious disease experts (ST and AC): Alpha, 12 January 2020 to 6 January 2021, Delta, 9 January 2021 to 12 January 2021, and Omicron, 2 January 2022 to 7 July 2022. The dates based on phylogenetic trees were retrieved from Nextstrain [13]. The intervals with the highest entropy residues were determined for each variant (see Appendix A). The Nextstrain is an online SARS-CoV-2 database that maps the evolution and epidemiology of the virus [14,15]. Additionally, the VHA COVID-19 shared data resources provided a subsample of genome sequencing for the SARS-CoV-2 virus. We observed high accuracy (Alpha, 99.2%; Delta, 97.5%; Omicron, 98.1%) and precision (Alpha, 91.3%; Delta, 96.9%; Omicron, 100.0%) between the sequenced samples and date-based samples (see Appendix A).

#### 2.2.2. Laboratory Biomarkers

The primary outcomes were laboratory biomarkers. We curated the first laboratory drawn from the patients during the hospitalization intervals to measure the laboratory biomarkers of interest. The laboratory biomarkers of interest were recommended by our medical advisory team (AS, STT, AC). We collected the following laboratory biomarkers using the Logical Observation Identifiers Names and Codes (LOINC) codes: CRP (‘11039-5’, ‘14634-0’, ‘1988-5’, ‘30522-7’, ‘35648-5’, ‘48421-2’, ‘71426-1’, ‘76485-2’, ‘76486-0’), Ferritin (‘14723-1’, ‘14724-9’, ‘20567-4’, ‘2276-4’, ‘24373-3’, ‘35209-6’), alanine aminotransferase (‘1742-6’, ‘1743-4’, ‘1744-2’, ‘44785-4’, ‘76625-3’, ‘77144-4’, ‘16325-3’, ‘1916-6’, ‘1742-6’, ‘1743-4’, ‘1744-2’, ‘44785-4’, ‘76625-3’, ‘77144-4’, ‘48134-1’, ‘16325-3), aspartate aminotransferase (‘1920-8’, ‘27344-1’, ‘30239-8’, ‘44786-2’, ‘88112-8’, ‘48136-6’, ‘54500-4’, ‘1916-6’). Lactate dehydrogenase (‘2546-0’, ‘2545-2’, ‘2547-8’, ‘2549-4’, ‘2548-6’, ‘49279-3’, ‘5910-5’, ‘42929-0’), and Albumin (‘1751-7’, ‘18180-0’, ‘2862-1’, ‘43712-9’, ‘54347-0’, ‘61151-7’, ‘61152-5’, ‘62234-0’, ‘62235-7’, ‘76631-1’, ‘77148-5’). If any laboratories fall outside the normal range, we considered it as abnormal or one. The secondary outcome was in-hospital mortality. The in-hospital mortality documented by the COVID-19 shared data resource.

#### 2.2.3. Other Variables

We used VHA Electronic Medical Record (EMR) to extract age (≥50 & <65, 65≥ & <75, ≥75 & <85, and ≥85 years), sex (male and female), BMI (categorized to <18.5, 18.5–30, and ≥30 kg/m^2^), race (White, Black, Others), Charlson Comorbidity Index (CCI), and frailty status. We used the validated VA frailty index (VA-FI) which is based on the accumulation of deficits framework and counts 31 age-related variables according to an established approach [16]. The VA-FI values were categorized as robust (≤0.1), prefrail (>0.1 and ≤0.2), and frail (>0.2). Any vaccination (any-Vax) referred to patients who received any dose of vaccines before the index date of hospitalization. 

### 2.3. Statistical Analysis

Mean and standard deviation were calculated for continuous outcomes, and count and percentage were calculated for categorical outcomes. We used logistic regression models to calculate the odds ratio (OR) of abnormal laboratories. We adjusted the OR (aOR) with age, sex, BMI, race, ethnicity, CCI, VA-FI, and vaccine record. We reported the ORs of in-hospital mortality between variants and adjusted them with age, sex, BMI, race, ethnicity, CCI, and VA-FI. 

We used the least absolute shrinkage and selection operator (LASSO) to identify the most important predictors of in-hospital mortality with 10-fold cross-validation. Then, we use multiple regression analysis to estimate the odds ratio. We forced the race variable to the multiple regression model. The regression was performed in those patients who had reported CRP laboratory biomarkers. The following variables were used as independent predictors: CRP, variants, age, gender, race, ethnicity, BMI, CCI, frailty status, and vaccination records. We reported odd ratios and 95 percent confidence intervals (95%CI) and Beta. The statistical significance was set at 2-sided *p* < 0.05. The statistical analyses were performed using IBM SPSS Statistics version 24 (IBM, Armonk, NY, USA) and R programming version 4.2.0 (R Foundation for Statistical Computing, Vienna, Austria).

## 3. Results

**Figure 1:**
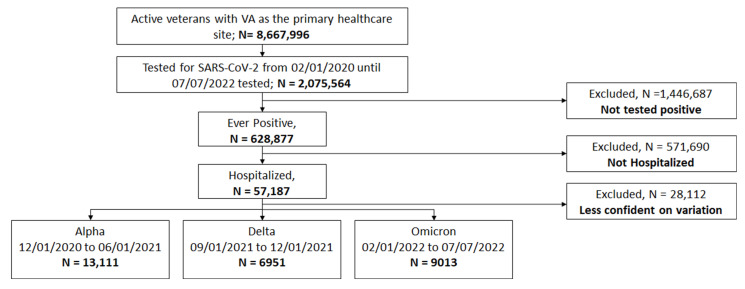
Strobe diagram of patient eligibility determinations for analysis.

We classified 13,111 as Alpha, 6951 as Delta, and 9013 as Omicron. We excluded 28,112 as they tested positive outside the concurred intervals for each variant, Figure 1, and during these excluded periods, multiple variants may be circulating. The percentage of patients with age ≥ 65 years in the Omicron (72.8%) was higher than Alpha (67.6%) and Delta (65.4%). The percentage of patients with BMI ≥ 30 kg/m^2^ was lower in Omicron (32.3%) compared to Alpha (44.2%) and Delta (43.5%). The percentage of patients with frail status was lower in Omicron (29.7%) compared to Alpha (58.3%) and Delta (47.4%). We reported the number of laboratory results available for patients for each variant, along with the median and interquartile intervals, Table 1. The median of CRP in the Omicron (8.7 mg/L) was lower than Alpha (12.6 mg/L) and Delta (13.3 mg/L). The median of Ferritin in Omicron (233.0 ng/mL) was lower than Alpha (432.9 ng/mL) and Delta (460.5 ng/mL). The same trend was observed in alanine aminotransferase, aspartate aminotransferase, and lactate dehydrogenase, Table 1.

The number of patients with abnormal laboratory findings are reported in Table 2. The percentage is calculated based on the total number of laboratories available in each variant group. The lowest percentage of abnormal CRP was observed in Omicron (80.0%) compared to Alpha (86.4%) and Delta (88.8%). The lowest percentage of abnormal ferritin was observed in Omicron (40.2%) compared to Alpha (61.4%) and Delta (63.6%). The same trend was observed in alanine aminotransferase, aspartate aminotransferase, lactate dehydrogenase, and albumin, Table 2. The adjusted odds of abnormal CRP among Alpha was 94% higher (aOR, 1.94, 95% CI: 1.75–2.15) compared to Omicron and among Delta 85% higher (aOR, 1.85, 95% CI: 1.64–2.09) compared to Omicron. The adjusted odds of abnormal ferritin among Alpha was 44% higher (aOR, 1.44, 95% CI: 1.30–1.60) compared to Omicron and among Delta 40% higher (aOR, 1.40, 95% CI: 1.24–1.58) compared to Omicron. The adjusted odds of abnormal alanine aminotransferase among Alpha were 41% higher (aOR, 1.41, 95% CI: 1.26–1.58) compared to Omicron and among Delta 25% higher (aOR, 1.25, 95% CI: 1.09–1.43) compared to Omicron. The adjusted odds of abnormal aspartate aminotransferase among Alpha were 53% higher (aOR, 1.53, 95% CI: 1.37–1.72) compared to Omicron and among Delta 36% higher (aOR, 1.36, 95% CI: 1.19–1.56) compared to Omicron. The adjusted odds of abnormal lactate dehydrogenase among Alpha were 69% higher (aOR, 1.69, 95% CI: 1.52–1.88) compared to Omicron and among Delta 79% higher (aOR, 1.79, 95% CI: 1.58–2.03) compared to Omicron. The adjusted odds of abnormal albumin among Alpha were 44% higher (aOR, 1.44, 95% CI: 1.26–1.63) compared to Omicron and among Delta 19% higher (aOR, 1.19, 95% CI: 1.02–1.38) compared to Omicron, Table 2. 

The lowest in-hospital mortality was observed in the Omicron (6.2%) compared to Alpha (11.2%) and Delta (13.5%) in Table 3 and Appendix B. The adjusted odds of in-hospital mortality were 1.68-times higher in Alpha (aOR, 1.68, 95% CI: 1.47, 1.91) compared to Omicron, and it was 1.92-time higher in Delta compared to Omicron (aOR, 1.92, 95% CI: 1.73, 2.12). The adjusted odds of 60-day follow-up mortality were 81% higher in Alpha (aOR, 1.81, 95% CI: 1.62, 2.02) compared to Omicron, and it was 2.04-time higher in Delta compared to Omicron (aOR, 2.04, 95% CI: 1.88, 2.23). The sensitivity analysis showed the same significant trend when we stratified the patients based on vaccination status, Table 3. 

The LASSO algorithm excluded race as the most important variable. We forced-fed the race variable to report the ORs. We observed that age ≥ 85 years (Beta, 2.16, OR, 8.64, 95% CI: 6.06, 12.32), age 75–85 (Beta, 1.74, OR, 5.73, 95% CI: 4.04, 8.12), age 65–75 (Beta, 1.40, OR, 4.06, 95% CI: 2.88, 5.74), Delta variant (Beta, 0.74, OR, 2.10, 95% CI: 1.83, 2.41), BMI < 18.5 (Beta, 0.58, OR, 1.78, 95% CI: 1.47, 2.15), Frailty Status-frail (Beta, 0.51, OR, 1.67, 95% CI: 1.44, 1.93) are the top predictors of in-hospital mortality, Table 4. The sensitivity analysis showed the same significant trend when we stratified the patients based on vaccination status, Table 4.

## 4. Discussion

We conducted a retrospective study using VHA administrative databases among veterans who tested positive for SARS-CoV-2 for the first time and were hospitalized in the VHA healthcare systems. We categorized patients into three groups, Alpha, Delta, and Omicron, based on the defined time intervals when each was the dominant circulating strain. We observed that Omicron patients showed significantly lower inflammatory profiles compared to Alpha and Delta variants of SARS-CoV-2. Further, mortality rates differed significantly among the three variants’ times, with the lowest mortality associated with the Omicron period. The findings persisted after stratifying for vaccination status. Among risk factors of in-hospital mortality, high chronicle age (≥65 years) had a similar expected change in log odds of mortality per unit change. Non-Omicron variants (i.e., Delta), BMI < 18.5, and being frail were the other risk factors for mortality with the same expected changes. Additionally, the CRP inflammatory biomarker was selected as one of the most important significant predictors of in-hospital mortality along with other factors.

COVID-19 results in two inflammatory phases, including direct virus-mediated tissue damage, followed by the second phase of local and systemic inflammation related to the host’s immune response [17]. The resulting inflammation may vary depending on the virus and the host and correlate with the spectrum and severity of the clinical presentation of the disease and related mortality [18]. Hui and colleagues showed that the Omicron variant replicates faster than other variants in the bronchi but less efficiently in the lung parenchyma [5]. Suzuki and colleagues showed that compared to Alpha and Delta, Omicron resulted in fewer pathological findings in the lungs of hamsters [19]. Data indicates that Omicron transmissibility, including breakthrough infections, was higher compared to other variants, and this correlated with deletions and mutations in its genetic structure [20,21]. Our data, consistent with the literature, show that patients admitted with COVID-19 during the Omicron dominant period had a lower mortality rate compared to those infected during the Alpha and Delta dominant periods [3,20,21,22,23,24,25]. Thus, clinical as well as in vivo and in vitro data suggest a decoupling between clinical presentation and transmissibility of Omicron when compared to Alpha and Delta variants. Our data clearly shows that systemic markers of inflammation are lower in Omicron compared to Alpha and Delta. Interestingly, even after adjustment for various factors, including the study variants, CRP significantly predicted higher mortality. This finding is novel and may stem from a combined effect of the inherent ability of a virus causing inflammation and the genetic composition of the host to mount inflammation.

Madhi and colleagues postulated that the cell-mediated immunity of prior COVID-19 partly explained the decoupling. Bhattacharyya and colleagues suggested that part of the reduced clinical severity of Omicron compared to Delta may stem from the stronger immune-evasion capability of Omicron compared to prior variants [26]. This strong immune evasion in turn allows Omicron to infect those with prior history of COVID-19 infection. A novel aspect of our study is that we enrolled individuals with first-time documented severe infection with the virus. Although prior infection and or vaccination offers some degree of protection against Omicron, even in first-time infected individuals, Omicron results in more favorable clinical outcomes than Delta and Alpha.

Studies show that COVID-19 vaccines significantly lower the incidence of clinically severe disease. Data from Altarawneh and colleagues showed that previous infection and or vaccination with BNT162b2 strongly reduced the rate of severe, critical, or fatal COVID-19 infection [27]. Our data is novel as it showed that the variances in mortality rates among the studied groups remained significant even in vaccinated patients. The data show that the adjusted ORs of in-hospital mortality is significantly higher than vaccinated patients. Thus, prior vaccination only to a mild degree explains the differences in the study outcomes between the Delta and Omicron groups.

The majority of the studies showing favorable clinical outcomes in Omicron versus prior variants are in population with age less than 65 [28]. Bhattacharyya also suggested that immunosenescence related to aging may explain worse clinical outcomes in Delta compared to Omicron [26]. Auvigne and colleagues reported that the lower severity of clinical illness in Omicron compared to Delta is less prominent in the elderly [29]. In our cohort, the average age in the three groups was similar, which makes the immunosenescence of less importance in our cohort. Krutikov and colleagues studied nursing home residents with a median age of 84.5 years and reported favorable outcomes, including hospitalization and mortality in Omicron compared to prior variants [30]. In our study as well as Krutikov and colleagues, the more favorable clinical outcomes in Omicron versus Alpha and Delta remained significant after adjusting for age, sex, prior infection, and vaccination status. To differentiate the differential mortality rates of the three studied variants, we used a validated index of frailty. Favorable mortality rates in Omicron compared to the others continued after adjusting for frailty status. 

Our team, as well as others, showed that mortality and poor clinical outcomes in COVID-19 are linked to the presence of comorbid conditions as assessed by Charlson Comorbidity Index [11,31]. Our data shows that prior to or after adjustment for comorbid conditions using CCI; mortality remains significantly lower in Omicron compared to the two other study variants. 

We used a large national cohort of patients from a healthcare system with an approximate distribution of race and ethnicity of the US population. Additionally, our cohort benefited from near-complete demographics and characteristics with up-to-date information retrievals. Importantly, our analysis is among the first and largest to compare the inflammatory laboratory biomarkers between three major SARS-CoV-2 variants in the US. Our study outcomes were limited to in-hospital mortality due to delays in reporting the out-of-hospital death reports. We also limited the number of reported laboratory biomarkers due to the fact that some patients may not have all the laboratory biomarkers of interest. Our analysis was limited to sets of variables available in the dataset, and we were not able to report on social determinants of health. Due to the large scale of this study, manual chart review was not feasible, and we are unable to determine the indications for SARS-CoV-2 testing and which symptoms, if any, the patients experienced.

## 5. Conclusions

In summary, our data established a close correlation among variants of SAR-CoV-2 variants, mortality, and severity of inflammation. Our clinical data support the notion that the lower immunopathogenesis of Omicron and, thus, lower produced inflammation is a major reason for a more favorable clinical outcome compared to Alpha and Delta variants. 

## Figures and Tables

**Table 1:** Demographics and clinical characteristics in the Alpha, Delta, and Omicron variation groups among veterans with COVID.

	Alpha	Delta	Omicron
N	13,111 (45.1)	6951 (23.9)	9013 (31.0)
Age: M (SD)	68.5(13.9)	67.6 (14.1)	70.1 (14.3)
Age 19–50, N (%)	1298 (9.9)	807 (11.6)	849 (9.4)
Age 50–65, N (%)	2955 (22.5)	1603 (23.1)	1603 (17.8)
Age 65–75, N (%)	4624 (35.3)	2409 (34.7)	2927 (32.5)
Age 75–85, N (%)	2708 (20.7)	1464 (21.1)	2387 (26.5)
Age ≥ 85, N (%)	1526 (11.6)	668 (9.6)	1247 (13.8)
Sex, Male, N (%)	12,418 (94.7)	6563 (94.4)	8542 (94.8)
Race, N (%)			
White	8421 (64.2)	5097 (73.3)	6375 (70.7)
Black	3461 (26.4)	1252 (18.0)	1825 (20.2)
Other	1229 (9.4)	602 (8.7)	813 (9.0)
Ethnicity-Hispanic, N (%)	1114 (8.5)	469 (6.7)	781 (8.7)
BMI, Kg/m^2^, M (SD)	29.8 (7.1)	29.5 (7.1)	28.0 (6.8)
BMI < 18.5, N (%)	372 (2.8)	233 (3.4)	414 (4.6)
BMI 18.5–30, N (%)	6939 (52.9)	3696 (53.2)	5688 (63.1)
BMI ≥ 30, N (%)	5800 (44.2)	3022 (43.5)	2911 (32.3)
Comorbid Conditions			
CCI, M (SD)	3.0 (2.7)	2.7 (2.6)	3.3 (2.9)
CCI ≥ 2, N (%)	8378 (63.9)	4069 (58.5)	6043 (67.0)
Frailty, M (SD)	0.3 (0.2)	0.2 (0.2)	0.1 (0.2)
Robust	2309 (20.2)	1462 (24.3)	3992 (60.7)
Prefrail	2469 (21.6)	1334 (22.1)	629 (9.6)
Frail	6669 (58.3)	3232 (53.6)	1952 (29.7)
Any vaccine record	1467 (11.2)	3298 (47.4)	6458 (71.7)
Laboratory Tests			
C-reactive protein, N (N = 14,916)	7570 (50.8)	4011 (26.9)	3335 (22.4)
C-reactive protein, mg/L, M(IQR)	12.6 (4.5, 47.1)	13.3 (5.1, 48.2)	8.7 (2.5, 31.2)
Ferritin, N (N = 13,859)	7073 (51.0)	3411 (24.6)	3375 (24.4)
Ferritin, ng/mL, M(IQR)	432.9 (206.8, 811.0)	460.5 (216.4, 926.4)	233.0 (109.0, 494.6)
Alanine aminotransferase, N (N = 20,923)	9897 (47.3)	5187 (24.8)	5839 (27.9)
Alanine aminotransferase, U/L, M(IQR)	25.0 (17.0, 39.0)	27.0 (18.0, 41.0)	20.0 (14.0, 31.0)
Aspartate aminotransferase, N (N = 20,884)	9918 (47.5)	5187 (24.8)	5779 (27.7)
Aspartate aminotransferase, U/L, M(IQR)	32.0 (22.0, 47.0)	34.0 (23.0, 51.0)	24.0 (18.0, 37.0)
Lactate dehydrogenase, N (N = 11,127)	5962 (53.6)	2807 (25.2)	2358 (21.2)
Lactate dehydrogenase, IU/L, M(IQR)	267.0 (199.0, 369.0)	284.0 (206.0, 406.0)	203.0 (161.0, 281.0)
Albumin, N (N = 23,159)	10,777 (46.5)	5766 (24.9)	6616 (28.6)
Albumin, g/dL, M(IQR)	3.2 (2.8, 3.5)	3.2 (2.8, 3.5)	3.3 (2.9, 3.6)

M (SD) = mean and standard deviation, M(IQR) = median and interquartile, CCI: Charlson Comorbidity Index.

**Table 2:** Comparing the abnormality after testing positive for COVID-19 among 3 variants groups: Alpha, Delta, and Omicron.

				Odds Ratio (95%Confidence Interval)			
Abnormality Status	Variation Status, N (%)			Alpha vs. Omicron		Delta vs. Omicron	
	Alpha	Delta	Omicron	Unadjusted	Adjusted ^†^	Unadjusted	Adjusted ^†^
Inflammatory Markers							
C-reactive protein	6540 (86.4)	3562 (88.8)	2669 (80.0)	2.29 * (2.14, 2.45)	1.94 * (1.75, 2.15)	2.30 * (2.18, 2.44)	1.85 * (1.64, 2.09)
Ferritin	4344 (61.4)	2168 (63.6)	1357 (40.2)	1.54 * (1.45, 1.65)	1.44 * (1.30, 1.60)	1.91 * (1.81, 2.02)	1.40 * (1.24, 1.58)
Liver Inflammation Markers							
Alanine aminotransferase	1701 (17.2)	996 (19.2)	853 (14.6)	1.49 * (1.38, 1.60)	1.41 * (1.26, 1.58)	1.60 * (1.50, 1.70)	1.25 * (1.09, 1.43)
Aspartate aminotransferase	3607 (36.4)	2212 (42.6)	1490 (25.8)	1.55 * (1.44, 1.67)	1.53 * (1.37, 1.72)	1.69 * (1.59, 1.80)	1.36 * (1.19, 1.56)
Lactate dehydrogenase	3796 (63.7)	1833 (65.3)	992 (42.1)	1.85 * (1.73, 1.98)	1.69 * (1.52, 1.88)	2.31 * (2.18, 2.45)	1.79 * (1.58, 2.03)
Liver Metabolic Function							
Albumin	6857 (63.6)	3685 (63.9)	3741 (56.5)	1.66 * (1.52, 1.80)	1.44 * (1.26, 1.63)	1.61 * (1.50, 1.73)	1.19 * (1.02, 1.38)

^†^ Results were adjusted by age, sex, body mass index, race, ethnicity, Charlson Comorbidity Index (CCI), VA frailty index, and vaccine record status. * The *p*-value < 0.05.

**Table 3:** Comparing in-hospital mortality among three variation groups: Alpha, Delta, and Omicron after the COVID-19 test.

	Variation Status, N (%)			Odds Ratio (95%Confidence Interval)			
	Alpha	Delta	Omicron	Alpha vs. Omicron		Delta vs. Omicron	
				Unadjusted	Adjusted ^†^	Unadjusted	Adjusted ^†^
In-hospital Mortality	1471 (11.2)	939 (13.5)	557 (6.2)	2.17 * (1.89, 2.49)	1.68 * (1.47, 1.91)	2.37 * (2.12, 2.65)	1.92 * (1.73, 2.12)
60-day follow-up Mortality	2214 (16.9)	1127 (16.2)	815 (9.0)	1.85 * (1.63, 2.08)	1.81 * (1.62, 2.02)	1.95 * (1.77, 2.14)	2.04 * (1.88, 2.23)
Stratifying Analysis based on vaccination records							
Mortality in any-Vax	2 (0.1)	348 (10.6)	374 (5.8)	NA	NA	1.92 * (1.65, 2.24)	1.49 * (1.21, 1.83)
60-day follow-up Mortality	5 (0.3)	432 (13.1)	559 (8.7)	NA	NA	1.59 * (1.39, 1.82)	1.27 * (1.06, 1.51)

^†^ Results were adjusted by age, sex, body mass index, race, ethnicity, Charlson Comorbidity Index (CCI), and VA frailty index. * The *p*-value < 0.05.

**Table 4:** Results of multivariate logistic regression for 14,916 patients with reported C-Reactive Protein laboratory results.

	All		Vaccinated	
	OR (95%CI)	Beta	OR (95%CI)	Beta
Abnormal CRP	1.24 (1.14, 1.36) *	0.22	1.26 (1.06, 1.50) *	0.23
Omicron	Reference	Reference	Reference	Reference
Delta	2.10 (1.83, 2.41) *	0.74	1.45 (1.18, 1.78) *	0.37
Alpha	1.61 (1.41, 1.84) *	0.48	NA	NA
Age < 50	Reference	Reference	Reference	Reference
age 50–65	1.99 (1.40, 2.85) *	0.69	0.94 (0.40, 2.18)	−0.07
age 65–75	4.06 (2.88, 5.74) *	1.40	1.81 (0.82, 4.00)	0.60
age 75–85	5.73 (4.04, 8.12) *	1.74	2.85 (1.30, 6.28) *	1.05
age ≥ 85	8.64 (6.06, 12.32) *	2.16	4.08 (1.84, 9.03) *	1.41
Sex-Male	1.52 (1.15, 2.00) *	0.42	2.57 (1.13, 5.86) *	0.94
Race-White	Reference	Reference	Reference	Reference
Race-Black	0.90 (0.80, 1.00)	−0.11	0.83 (0.65, 1.07)	−0.18
Race-Other	1.06 (0.91, 1.24)	0.06	0.86 (0.60, 1.21)	−0.15
Ethnicity				
BMI 18.5–30	Reference	Reference	Reference	Reference
BMI < 18.5	1.78 (1.47, 2.15) *	0.58	2.50 (1.82, 3.44) *	0.92
BMI ≥ 30	0.98 (0.90, 1.08)	−0.02	0.83 (0.68, 1.02)	−0.18
CCI ≥ 2	1.11 (1.00, 1.23) *	0.10	1.56 (1.21, 2.01) *	0.45
Frailty Status -Robust	Reference	Reference	Reference	Reference
Frailty Status -prefrail	1.61 (1.37, 1.89) *	0.48	1.21 (0.85, 1.74)	0.19
Frailty Status -frail	1.67 (1.44, 1.93) *	0.51	1.83 (1.44, 2.33) *	0.60

OR (95%CI) = odds ratio and 95 percent confidence intervals, CRP = reported C-Reactive Protein laboratory, BMI = body Mass Index, CCI = Charlson Comorbidity Index. * The *p*-value < 0.05.

**Figure A1:**
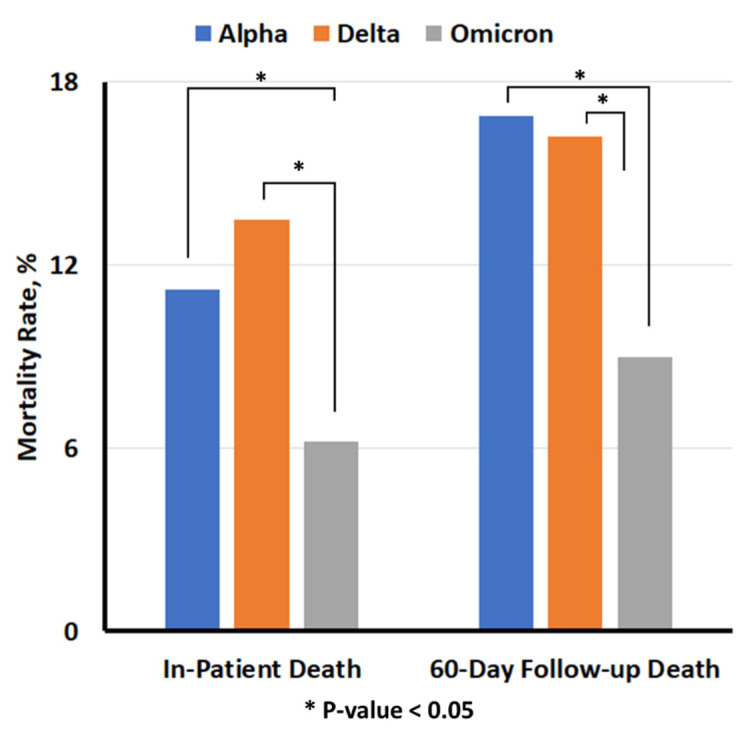
In-hospital mortality among three variation groups: Alpha, Delta, and Omicron after the COVID-19 test.

## Data Availability

The data are available behind the VHA firewall, and they cannot leave the VHA electronic health records. Any request for data access requires official approval process.

## Appendix A

We determined the SARS-CoV-2 variants based on the time intervals that our team of clinicians provided us. The time intervals determined based on the based on phylogenetic trees were retrieved from Nextstrain [13]. To ensure confidence in the variant, we used the COVID-19 shared data resource (CSDR) in the VHA, where they reported the results of genetic sequence for the subsample of the SARS-CoV-2 virus. According to the CSDR, there were 2670 samples. Table A1 shows the distribution of samples based on one date versus the genetic sequences.

**Table A1:** Reporting the number of cases grouped into three SARS-CoV-2 variants based on the genetic sequencing versus the date.

		Variant Determined Based on the Genetic Sequencing			
		Alpha	Delta	Omicron	Total
Variant determined based on the date	Alpha	42	2	2	46
	Delta	18	2031	48	2097
	Omicron	0	0	527	527
	Total	60	2033	577	2670

According to Table A1, we reported the precision, negative predictive value (NPV), sensitivity, specificity, and accuracy for each variant based on the one-versus-rest approach (see Table A2) [32].

**Table A2:** Reporting the performance of the interval-based method for identifying SARS-CoV-2 variants versus the genetic sequencing on subsample 2670 cases.

	Alpha	Delta	Omicron
Precision	0.913	0.969	1.000
Negative Predictive Value	0.993	0.997	0.977
Sensitivity	0.700	0.999	0.913
Specificity	0.998	0.896	1.000
Accuracy	0.992	0.975	0.981
